# Important biological information uncovered in previously unaligned reads from chromatin immunoprecipitation experiments (ChIP-Seq)

**DOI:** 10.1038/srep08635

**Published:** 2015-03-02

**Authors:** Wilberforce Zachary Ouma, Maria Katherine Mejia-Guerra, Alper Yilmaz, Pablo Pareja-Tobes, Wei Li, Andrea I. Doseff, Erich Grotewold

**Affiliations:** 1 Molecular, Cellular and Developmental Biology (MCDB) Graduate Program, The Ohio State University, Columbus, OH, USA; 2 Center for Applied Plant Sciences (CAPS), The Ohio State University, Columbus, OH, USA; 3 Department of Bioengineering, Yildiz Technical University, Istanbul, Turkey; 4 Oh no sequences! Research group, Era7 Information Technologies SLU, Granada, Spain; 5 Department of Molecular Genetics, The Ohio State University, Columbus, OH, USA; 6 Department Physiology and Cell Biology, Heart and Lung Research Institute, The Ohio State University, Columbus, OH, USA

## Abstract

Establishing the architecture of gene regulatory networks (GRNs) relies on chromatin immunoprecipitation followed by massively parallel sequencing (ChIP-Seq) methods that provide genome-wide transcription factor binding sites (TFBSs). ChIP-Seq furnishes millions of short reads that, after alignment, describe the genome-wide binding sites of a particular TF. However, in all organisms investigated an average of 40% of reads fail to align to the corresponding genome, with some datasets having as much as 80% of reads failing to align. We describe here the provenance of previously unaligned reads in ChIP-Seq experiments from animals and plants. We show that a substantial portion corresponds to sequences of bacterial and metazoan origin, irrespective of the ChIP-Seq chromatin source. Unforeseen was the finding that 30%–40% of unaligned reads were actually alignable. To validate these observations, we investigated the characteristics of the previously unaligned reads corresponding to TAL1, a human TF involved in lineage specification of hemopoietic cells. We show that, while unmapped ChIP-Seq read datasets contain foreign DNA sequences, additional TFBSs can be identified from the previously unaligned ChIP-Seq reads. Our results indicate that the re-evaluation of previously unaligned reads from ChIP-Seq experiments will significantly contribute to TF target identification and determination of emerging properties of GRNs.

Chromatin immunoprecipitation (ChIP) followed by high-throughput sequencing (ChIP-Seq) allows the *in vivo* characterization of genome-wide maps of protein-DNA interactions and epigenetic modifications. Given its advantages in coverage and resolution, ChIP-Seq has become the preferred approach to identify genome-wide binding sites of transcription factors (TFs) and RNA polymerase components for the ENCODE^1^ and modENCODE^2^ projects.

ChIP-Seq involves several experimental steps that start with the chemical crosslinking of proteins to DNA and culminates with the generation of millions of short sequence reads (hereafter referred to as “reads”)^3^ which are the input for computational analysis pipelines (Fig. 1). The first step in the analysis is the mapping of reads to a reference genome. Pre-processing steps that include quality checks are sometimes performed; however in most cases alignment programs make use of the whole dataset of raw reads. The result is an alignment file, and two sets of rejected reads: One that comprises unaligned reads, and the other formed by reads that map to multiple genomic locations, hereafter referred to as “multi-reads”. The next step in the analysis involves the discovery of enriched regions, usually represented as “peaks” corresponding to Transcription Factor Binding Sites (TFBSs). Almost all software applications use only reads aligning uniquely to the reference genome for the discovery of TFBSs^4^,^5^. However, the provenance of unaligned reads and to what extent they contain significant information that contributes to understanding TF function has not been studied.

Recently, the ENCODE and modENCODE projects established standardized guidelines for ChIP-Seq experiments and data analysis^5^. Additionally, experiments to generate datasets with benchmarking purposes have been carried out to identify the influence of sequencing depth in ChIP-Seq data^6^. From the analytical point of view, most of the evaluation has focused on the enrichment analysis and evaluation of different algorithms and their implementations for analysis. However, all the sources of bias affecting results from ChIP-Seq data are far from being characterized. In this regard, expanding exploration to different portions of the data and/or including information from a wide group of techniques, organisms, and analysis pipelines is likely to uncover other limitations associated with ChIP-Seq experiments.

A limitation of ChIP-Seq data is that it is usually characterized by a very large portion (20%–90%) of reads that fail to align to the corresponding reference genome (hereafter referred to as low read mappability), a phenomenon that has been observed in ChIP-Seq experiments ranging from humans to plants^7^,^8^,^9^,^10^,^11^,^12^. This is problematic because it decreases the amount of usable reads to sometimes as low as 20% of the total reads obtained, affecting the likelihood of identifying TFBSs and possibly resulting in conclusions bias by this information. Whereas most studies have focused on improving experimental procedures or software pipelines for the analysis of ChIP-Seq data^5^,^6^,^13^, the problem of large proportions of unaligned reads in ChIP-Seq studies has so far largely been ignored. Here, we investigate the origin and characteristics of the unaligned reads that characterize ChIP-Seq experiments using data available from different organisms. Applying a metagenomics-like approach, we investigated the provenance of reads failing to map to reference genomes in ChIP-Seq experiments from human (*Homo sapiens*), the fruit fly (*Drosophila melanogaster*), the roundworm (*Caenorhabditis elegans*), the thale cress (*Arabidopsis thaliana*), and maize (*Zea mays*). The analyses of over 4.3 × 10^10^ bp of raw sequence reads obtained from ChIP-Seq experiments deposited to the NCBI's Short Read Archive (SRA) showed that success of alignment is not specific to any particular ChIP-Seq run, experiment or project, since there is considerable variation in the amount of reads that align to the reference genome even in biological replicate ChIP-Seq experiments. Taxonomical classification of unaligned reads to their respective Operational Taxonomic Units revealed dual origin of unaligned reads in ChIP-Seq experiments: I) Genome-derived potentially legitimate sequences with mappable properties, and II) foreign DNA likely representing contamination of unaligned ChIP-Seq sequences with other taxonomic groups. Indeed, an in-depth ChIP-Seq analysis of one such potentially legitimate human reads dataset resulted in identification of novel TAL1 binding sites. These findings are important because the use of legitimate previously unaligned reads in identifying additional TFBSs results in the discovery of new target genes that could enhance construction of gene regulatory grids and networks.

## Results

### Read mappability varies across ChIP-Seq runs and experiments

Establishing gene regulatory network (GRN) architecture implies the integration of ChIP-Seq datasets usually obtained from different data sources, including data from multiple laboratories, different researchers within one laboratory and different TFs studies by the same researcher. These datasets result from biological and/or technical experimental and control replicates. As a result, an experiment aimed at determining TFBSs of a specific TF will have multiple sequencing files. Controls include one or more of the following: (i) Non-immunoprecipitated genomic DNA, commonly referred to as input DNA; (ii) DNA obtained by immunoprecipitation of chromatin from an individual lacking the particular TF; or (iii) DNA obtained by a mock immunoprecipitation of genomic DNA by an idiotypic antibody control, such as immunoglobulin G (IgG). In our analysis, we refer to data from an experiment as a set of ChIP-Seq datasets obtained from one or multiple ChIP-Seq runs carried out for determination of one TF binding profile. Occasionally, a single transcriptional regulatory study could aim to discover TFBSs for more than one TF.

**Figure 2:**
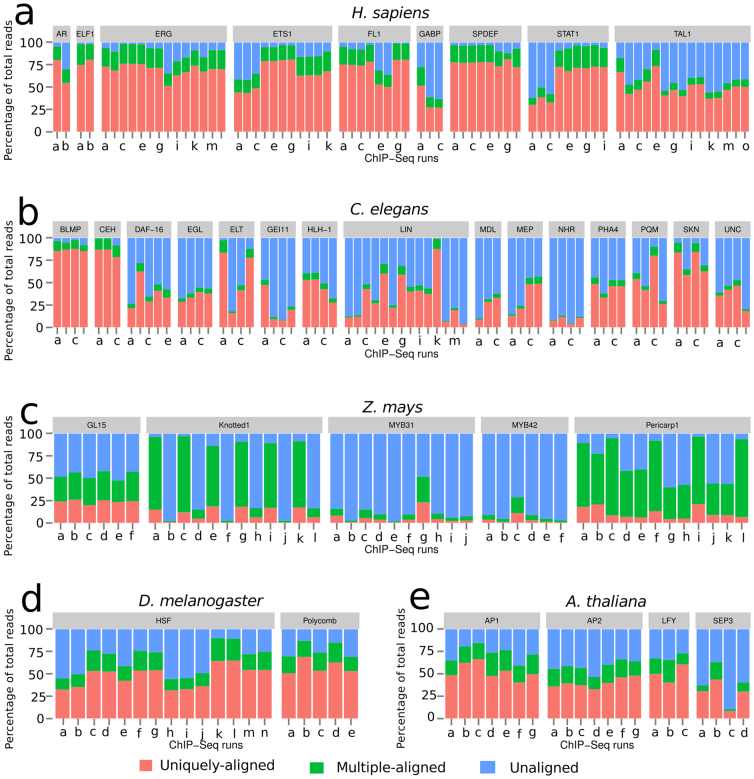
Proportions of uniquely-aligned, multiple-aligned, and unaligned reads in ChIP-Seq datasets of five organisms. Each bar represents a ChIP-Seq run; several runs constitute an experiment for determining binding patterns of one transcription factor. Runs have been grouped into their respective TFs indicated in the gray boxes; each run has been coded by an identifier representing NCBI Short Read Archive accession number for a ChIP-Seq run (See Supplementary File 1 for the accession numbers corresponding to each identifier in the figure).

Next, preprocessed reads exhibiting high sequence quality (Supplementary Fig. S2) were aligned to their respective reference genomes using Bowtie version 0.12.7^14^. This served two purposes: (i) To obtain unaligned reads for further analyses; and (ii) to observe variations in the numbers of both aligned and unaligned reads across different ChIP-Seq runs, experiments and organisms. Consistent with what was reported for each of the TFs (see Supplementary File 1 for the complete list of TFs), we observed large variations in the proportions of aligned reads within and between different ChIP-Seq experiments in sequence datasets of *H. sapiens*, *C. elegans*, *Z. mays*, *D. melanogaster*, and *A. thaliana* (Fig. 2, panels a, b, c, d and e respectively). These results suggest that no experiment for a particular TF had superior alignment proportions. Not surprisingly, given the highly repetitive nature of the maize genome^15^,^16^,^17^ and the still incomplete maize genome sequence, experiments in this species exhibited significantly lower proportions of uniquely-aligned reads, with the highest percentage being just slightly above 20% of the total reads (Fig. 2c). Interestingly however, we observed a high number of reads that aligned to multiple genomic regions in maize datasets, most of these are coming from the input control ChIP-Seq runs (Supplementary Fig. S1c). In contrast, percentages of uniquely-aligned reads in *Arabidopsis thaliana* experiments (Fig. 2e) were comparatively twice as much as maize (Fig. 2c); and percentages of multi-reads relatively lower, although variation in alignment proportions was still observed across different *A. thaliana* ChIP-Seq experiments.

Variations in the proportion of aligned reads within and between experiments were also observed for human (Fig. 2a), *C. elegans* (Fig. 2b), and *D. melanogaster* ChIP-Seq runs (Fig. 2d). Unlike plant ChIP-Seq runs, most human ChIP-Seq runs had more than 60% of their reads aligning uniquely to the human reference genome. In addition, all the human control input runs had around 60% of the total reads aligning uniquely with very little variation across different runs (Supplementary Fig. S1a). Consistent with a lower genome complexity (less repetitive DNA), *C elegans* datasets exhibited the least proportions of multi-reads, albeit with higher variations in uniquely-aligned reads within ChIP-Seq experiments (Fig. 2b). Similarly, *D. melanogaster* alignments exhibited very significant variations (Fig. 2d).

We also investigated the influence of read-length on mapping by systematically evaluating mappability of reads of varying lengths. Reads from *A. thaliana* and *D. melanogaster* ChIP-Seq runs were iteratively trimmed to obtain 20, 25, 30, 35 and 40 bp reads, resulting in five short read files for each run. The trimmed reads were realigned to their respective reference genomes to determine proportions of aligned, multiple-aligned and unaligned reads. In all the ChIP-Seq runs investigated, 20 bp reads exhibited an increase in the percentage of uniquely-aligned reads, ranging from approximately 2% to as high as 12% (Supplementary Fig. S3) (*p* -value < 0.001 for all pairwise Chi-square tests for equality of proportions between 20 bp uniquely- aligned proportions and each of the 25 bp, 30 bp, 35 bp and 40 bp uniquely-aligned proportions, for each ChIP-Seq run). Increased read length after 25 bp does not alter the proportion of aligned reads. However, and suggesting that using 20 bp reads is not a good idea, there is a significant increase in the percentage of multiple-aligned reads in the 20 bp ChIP-Seq runs compared to runs with longer reads, for the two genomes (*p* -value < 0.001 for all pairwise Chi-square tests for equality of proportions between 20 bp multiple-aligned proportions and each of the 25 bp, 30 bp, 35 bp and 40 bp multiple-aligned proportions, for each ChIP-Seq run). To investigate the influence of sequencing errors on mappability, we reanalyzed *A. thaliana* and *D. melanogaster* ChIP-Seq reads by performing alignments using Bowtie, first without any mismatch and then realigning the same set of reads while increasing the number of allowed mismatches from 1 to 3 (Bowtie allows a maximum of 3 mismatches), resulting in four alignment results for each ChIP-Seq run. As depicted in Supplementary Fig. S4, we observed a modest increase in the proportion of aligned reads with increasing number of allowed mismatches. However, there were two ChIP-Seq runs in *A. thaliana* (SRR070382 and SRR070384) and three runs in *D. melanogaster* (SRR039092, SRR039093 and SRR039096) that exhibited significant reduction of uniquely-aligned reads when mismatches were not allowed in the alignment process. Therefore, in order to account for the influence of sequencing errors during the alignment process, at least one mismatch must be allowed.

### Effect of sequence quality and GC content on read mappability

A correlation between quality of sequencing libraries and read mappability has been observed in *A. thaliana*^8^. Similarly, foreign contaminants introduced during the generation of sequencing libraries, especially at the adapter ligation step, as well as sequencing technology biases have been suggested to play a role in decreasing the number of reads aligning to reference genomes. To establish whether the variability in mappability is a consequence of variations in the quality of the sequences produced, we compared the sequence quality of aligned and unaligned reads using FASTQC, a sequence quality determination tool for high-throughput sequence data. Different from what was previously reported for *A. thaliana* ChIP-Seq experiments^8^,^13^ we observed similar sequence quality distributions between aligned and unaligned read datasets (Supplementary Fig. S2). Moreover, we did not observe any correlation between the proportion of unaligned reads and the total number of reads in a ChIP-Seq run (Supplementary Fig. S5). When we determined the GC content of the different genomes analyzed (Supplementary Table S1), we did not observe any correlation between GC content and proportion of aligned reads. As an illustration, *D. melanogaster* and *A. thaliana* have different GC content (41.51% and 36.05% respectively, Supplementary Table S1), but their alignment proportions are generally similar (Fig. 2d and Fig. 2e). Additionally, we explored the possibility of a correlative relationship between the age of the study or of the sequencing technology with the percentage of mapped reads. We did not observe any correlation between the sequencing technology and the percentage of mapped reads in the datasets that we analyzed. As an illustration, varying proportions of unique alignments in *C. elegans* (Figure 2c) can be observed in spite of one type of NGS technology having been used in sequencing the reads (Illumina Genome Analyzer, Supplementary File 1, Sheet 4). Variations in proportions of unaligned reads were also observed in datasets generated by Illumina Genome Analyzer II, for instance in human STAT1 and TAL1 short read datasets (compare Fig. 2a and Supplementary File 1 Sheet 2). These observations therefore suggest that the failure of reads aligning to their respective genomes is independent of dataset size, GC content, or the sequencing technology.

### Unaligned reads contain foreign DNA material

**Figure 3:**
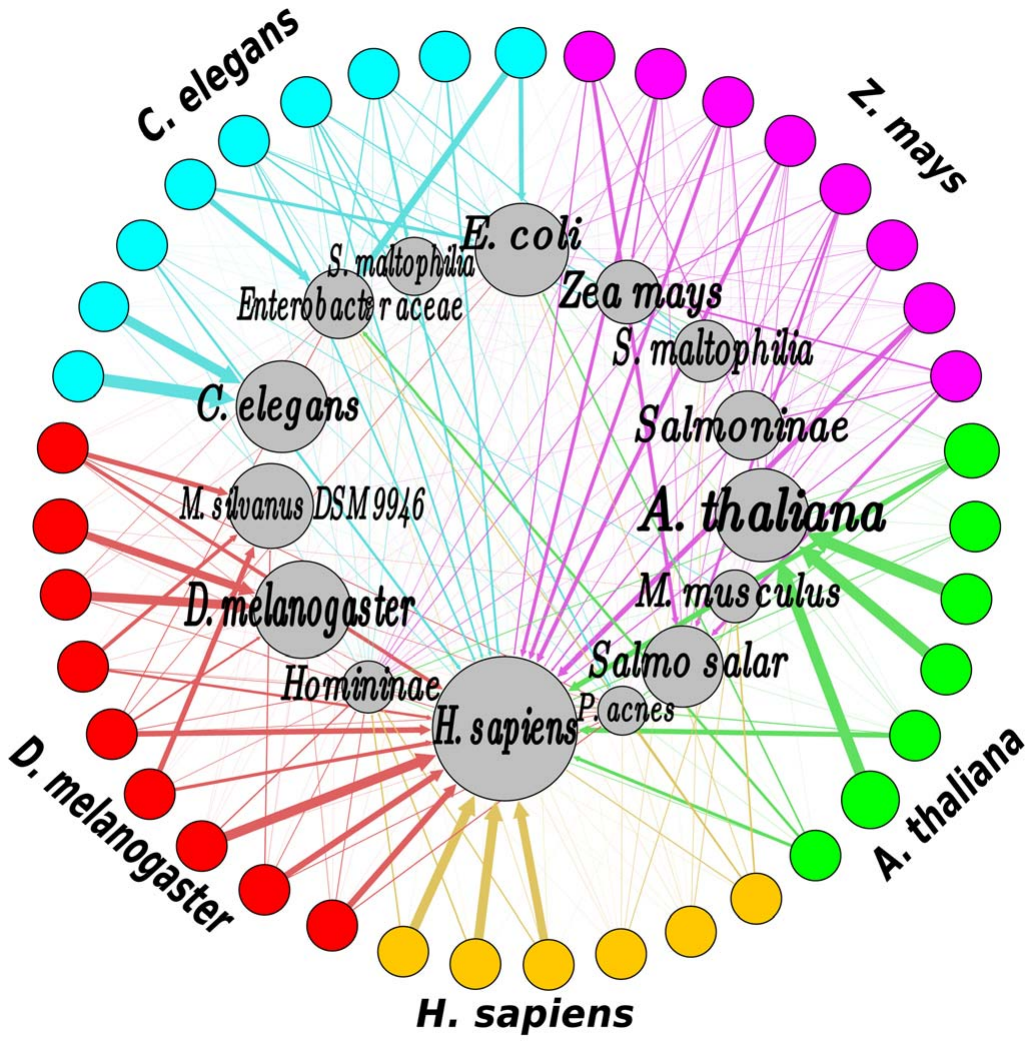
Taxonomic classification of unaligned reads reveals presence of potentially-legitimate reads as well as contaminant sequences. Inner circles represent taxonomic units, sizes reflect relative abundance of the taxonomically-classified reads in each unaliged ChIP-Seq dataset; outer circles represent unaligned ChIP-Seq datasets; edge thickness corresponds to the proportion of reads assigned to the corresponding taxonomic unit.

Since the number and complexity of the unaligned reads in all the datasets investigated here made it impossible to establish the origin of each read individually, we clustered unaligned reads (and for comparison purposes, aligned reads were also included) based on sequence similarity to obtain representative sequences for nucleotide database searches and subsequent taxonomic classification. To determine the provenance of the unaligned reads, we employed a nucleotide database search approach. Representative reads from each cluster of unaligned datasets were subjected to NCBI nucleotide database search using a Basic Local Alignment Search (BLAST) tool. Using each of the BLAST outputs, reads were classified into taxonomic units based on their BLAST hits and NCBI taxonomy tree. Assignment of unaligned reads into higher rank taxonomic units revealed the presence of three distinct groups of “Bacteria”, “Metazoa” and “Plantae” (Supplementary Fig. S6). Sequences derived from organisms other than that from which the chromatin was obtained (‘contaminants’) were found in appreciable amounts in the published datasets for the five organisms analyzed. Most strikingly, an experiment from the ENCODE project aimed at identifying genome-wide binding pattern of TAL1^18^ transcription factor had considerable amounts of reads in their ChIP-Seq runs assigned to the bacterial taxonomic unit, with some ChIP-Seq runs having as much as 60% of the unaligned reads being classified as bacterial sequences. In-depth analysis of the unaligned reads revealed predominant presence of three different species of bacteria: *Escherichia coli*, *Propionibacterium acnes* and other Enterobacteriacea (Fig. 3). Additionally, mice sequences were identified in two (accession numbers SRR070251 and SRR070252) of the human TAL1 ChIP-Seq datasets. Bacterial contamination from *Meiothermus silvanus* were also observed in Drosophila Heat Shock Factor (HSF) binding datasets (accessions SRR039095, SRR039099, SRR039100). Finally, the *C. elegans* DAF-16 datasets (SRR017602, SRR017605) contained significant amounts of both *E. coli* and Enterobacteriaceae sequences (Fig. 3).

### Presence of potentially legitimate reads in previously unaligned reads

**Figure 4:**
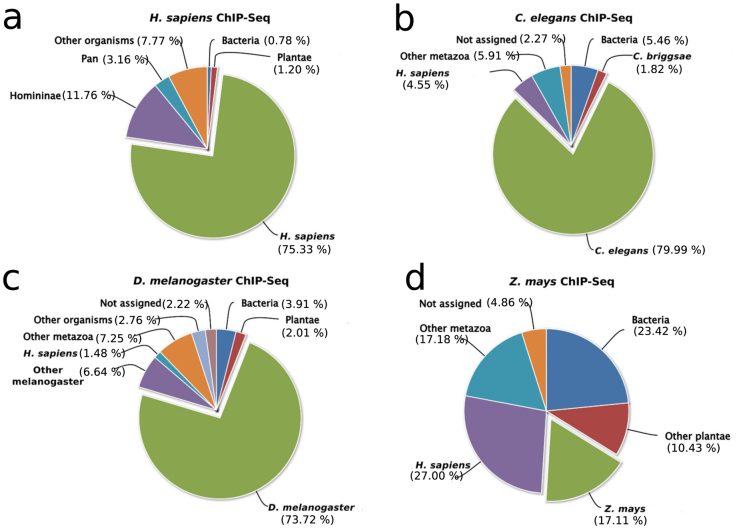
Taxonomic classification of previously unaligned reads datasets having potentially legitimate reads. Unaligned reads from selected *H. sapiens* (panel a), *C. elegans* (panel b), *D. melanogaster* (panel c) and *Z. mays* (panel d) ChIP-Seq runs were taxonomically classified to different taxonomic units.

Apart from uncovering potential contaminant sequences in the unaligned reads datasets, the taxonomic classification analyses revealed the presence of potentially legitimate reads. Human unaligned datasets, for instance, exhibited varying proportions of metazoan-derived sequences. In an ENCODE study^1^ aimed at determining the DNA-binding profiles of human Erythroblast Transformation Specific (ETS) family of transcription factors ERG, ELF1, SPI1 and SPDEF, approximately 70% of the unaligned reads in each run were assigned to the metazoan taxonomic unit, suggesting the presence of potentially legitimate human-derived reads (Supplementary Fig. S6). Indeed, a much closer examination involving assignment of reads into lower rank taxonomic units revealed the presence of significant amounts of human reads in these datasets, with up to 75% of the total previously unaligned reads being classified as sequences of human origin in selected datasets (Fig. 4a). Similarly, a significant proportion of previously unaligned reads in selected ChIP-Seq runs from *C. elegans* (80%), *D. melanogaster* (74%), and *Z. mays* (17%) were of potentially genome-derived origin, rather than contaminants or sequencing artifacts (Fig. 4 panels ‘b’, ‘c’ and ‘d’ respectively).

To investigate whether potentially legitimate reads correspond to true reads with mappable properties, we realigned the potentially legitimate reads to their respective reference genomes using SHRiMP^19^. Between 40% and 50% of previously unaligned and potentially-legitimate reads (already taxonomically classified reads from selected experiments) were mappable. Therefore, since the average percentage of previously unaligned reads that was classified in the same taxonomic unit as the species on which ChIP-Seq was performed was about 75% for human (realignment and validation was performed on human ChIP-Seq datasets), this translates to 30%–37.5% of the previously unaligned reads being mappable. We used SHRiMP instead of Bowtie in the realignment process because SHRiMP performs better for the mapping of short reads in highly polymorphic regions of the genome, and also in the handling of insertion-deletions (indels)^19^.

### Sensitivity analysis of commonly used next generation sequencing aligners

To determine the ability of different sequencing programs to align ChIP-Seq reads in a high throughput fashion, we evaluated different aligners on simulated reads from the maize genome (B73 RefGen_v2). A receiver Operating Characteristic (ROC) curve to determine performance of four alignment programs Stampy (http://www.well.ox.ac.uk/project-stampy), BWA (http://bio-bwa.sourceforge.net/), Novoalign (http://www.novocraft.com/main/page.php?s=novoalign) and Bowtie2 showed that Novoalign performed better than the rest, while Bowtie2 performed the least (Supplementary Fig. S7). Overall, SHRiMP was the most sensitive aligner, with sensitivity of 0.84. Bowtie version 0.12.7 sensitivity was 0.40. SHRiMP's superior sensitivity of 0.84 compared to other commonly used next generation sequencing aligners highlights its ability to remap previously unaligned reads.

### Identification of new binding peaks for human TAL1

**Figure 5:**
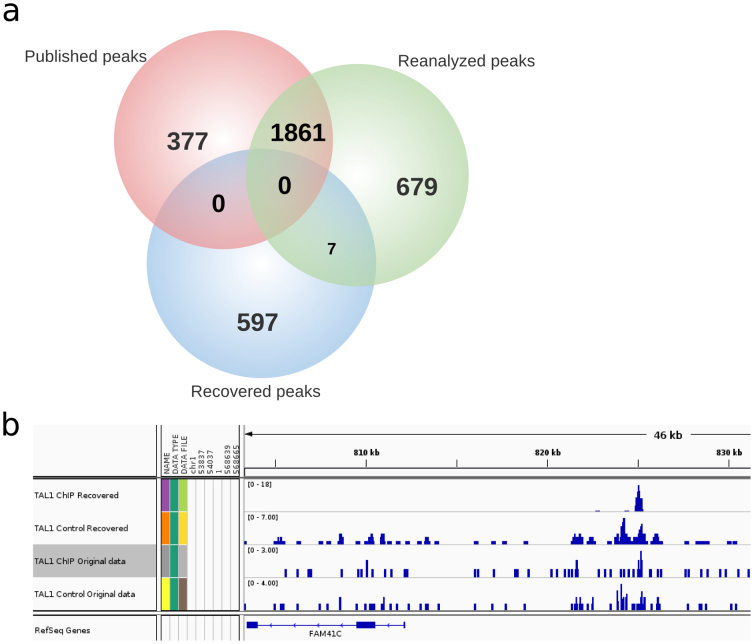
Venn diagram representation of overlap of published, reanalyzed and recovered peaks (a). Peak representation for a novel TAL1 binding site found by reanalysis of previously unaligned reads (b). ‘TAL1 ChIP Recovered’ and ‘TAL1 Control Recovered’ denote the ChIP and control tracks for the recovered peaks respectively; ‘TAL1 ChIP Original data’ and ‘TAL1 Control Original data’ denote the respective ChIP and control of the original TAL1 ChIP-Seq data before read recovery.

To test the hypothesis that previously unaligned reads datasets contain legitimate reads with mappable properties needed for identification of novel TFBSs, we set out to identify new peaks from the previously unaligned reads. We term these new peaks “recovered peaks”. We first obtained potentially legitimate reads which had been taxonomically classified as *H. sapiens* reads from TAL1 ChIP-Seq experiments and realigned them to the human reference genome using SHRiMP^19^. The choice of TAL1 ChIP-Seq for identification and experimental validation of novel binding sites was due to availability of antibodies used in the original study, allowing us to carry out an experimental validation. Peak-calling analysis of TAL1 potentially legitimate reads resulted in the identification of 604 new peaks (hereafter referred to as ‘recovered peaks’) by MACS (version 1.4.2)^20^ with a *p*-value cut-off of 0.005. In parallel, we performed the reanalysis of the raw TAL1 ChIP-Seq reads (SRA accession number SRX029597) by first aligning all the preprocessed TAL1 reads to the human reference genome using Bowtie, followed by identification of 2,547 peaks (hereafter referred to as reanalyzed peaks) using MACS (version 1.4.2) with a *p* -value cut-off of 0.005. The comparison between the previously published peaks obtained in the original TAL1 study (hereafter referred to as ‘published peaks’)^18^ (GEO accession number GSM614003) and the reanalyzed peaks showed an overlap of 1,861 peaks out of the 2,238 peaks published, in addition to 679 novel peaks that the reanalysis identified, which were not present among the published peaks (Fig. 5a).

Interestingly, however, the 604 recovered peaks (from the previously unaligned reads) overlapped very little with the reanalyzed (7 peaks overlap) and failed to overlap with the published peaks (Fig. 5a), in spite of the recovered peaks exhibiting a typical ChIP-Seq peak profile characteristic (Fig. 5b). This demonstrates that the recovered peaks were located in novel genomic regions not previously identified as TAL1 binding sites. Indeed, a positional analysis of peaks using the BEDtools suite (https://code.google.com/p/bedtools/) indicated that the peaks obtained from the recovered reads were positionally different from the published or reanalyzed peaks. These results indicate that newly-recovered peaks from previously unaligned reads correspond to regions of the genome distinct from those in the published and reanalyzed categories. Additionally, a motif enrichment analysis using the *de novo* motif discovery tool MEME (http://meme.nbcr.net/meme/) to identify enriched motifs in published, reanalyzed and recovered peaks in TAL1 data sets revealed previously identified TAL1 recognition motifs GATA and RUNX in the published peaks^18^, and TEAD, an unknown motif enriched in the recovered peaks, suggesting novelty of the recovered TAL1 peaks (Supplementary Fig. S8).

**Figure 6:**
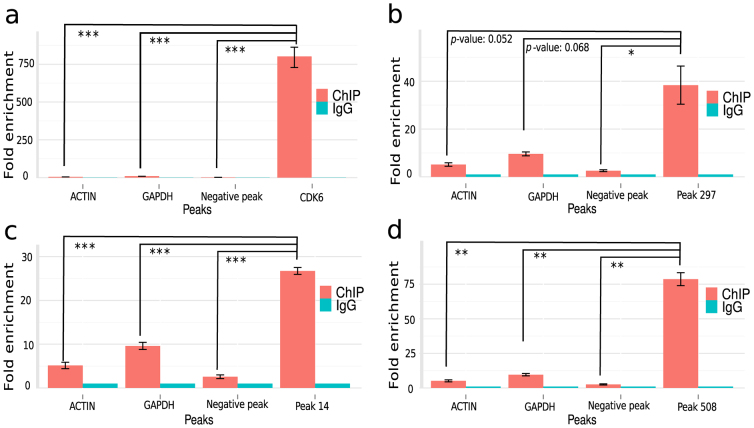
Validation of recovered TAL1 binding peaks by ChIP-qPCR. Each bar represents fold enrichment of peaks relative to IgG. Error bars represent standard error. Statistical significance is as a result of t-test between the validated peak enrichment and the negative controls of ACTIN, GAPDH, and negative peak; n = 3. Actual p-value is indicated when >0.05, otherwise p-value < 0.05 indicated by *; <0.01 by **; and <0.001 by ***. The CDK6 bar graph represents the results with a positive control; a known TAL1 direct target.

To experimentally evaluate whether the TAL1 target sequences identified corresponded to additional biological roles for TAL1, we conducted ChIP experiments followed by quantitative PCR (ChIP-qPCR) on Jurkat cells using commercially available TAL1 antibodies and 11 randomly selected peaks (see below). As positive controls, we used Cd69 and CdK6, which were previously shown to be TAL1 targets. Both of these genes showed statistically significant (*p* -value < 0.001, two-sided t-test) ChIP-Seq fold enrichment (over the input), when compared with the non-TAL1 targets ACTIN or GAPDH (Fig. 6a and Supplementary Fig. S9a). We randomly selected 11 of the peaks recovered from previously unaligned reads for validation by ChIP-qPCR, from which two peaks showed significant enrichment when compared to non-TAL1 targets (Fig. 6c and Supplementary Fig. S9c; *p* -value < 0.001, two-sided t-test). Additionally, three peaks showed significant fold enrichment with two-sided t-test p -value < 0.01 (Figure 6d; Supplementary Fig. S9b, f) and two peaks with two-sided t-test *p* -value < 0.05 (Fig. 6b; Fig. 5g). One peak showed suggestive but inconclusive evidence for fold enrichment when compared to a negative control peak (Supplementary Fig. S9d, two-sided t-test *p* -value of 0.077), while one peak showed non-significant enrichment compared to non-TAL1 targets (Supplementary Fig. S9e). Thus, out of the 11 randomly-selected newly identified peaks, eight showed a significant enrichment for TAL1 in ChIP experiments, suggesting that TAL1 binds to these genomic areas *in vivo*.

**Figure 7:**
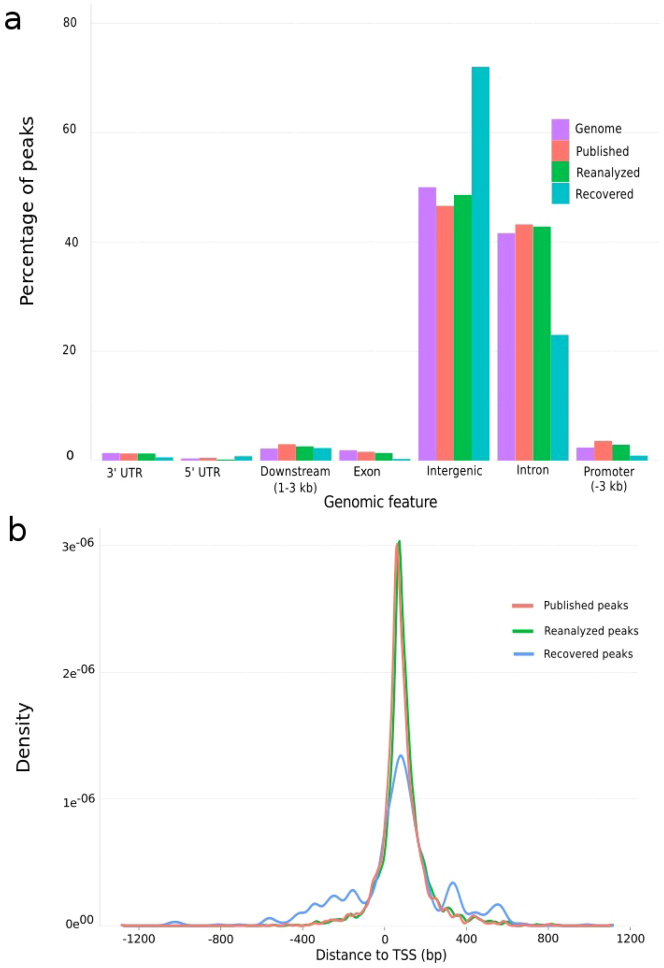
Peak distribution. (a) TAL1 binding regions relative to the human genome structure (purple color): Proportions of peaks were determined in genomic components of 3′ untranslated region (UTR), 5′ UTR, downstream, exon, intergenic, intron and promoter in published (red), reanalyzed (green) and recovered (blue) datasets. (b) Peak density relative to TSS: Kernel density distribution of peaks from the three categories of datasets exhibited a local enrichment around TSS.

To establish whether the peaks obtained from the recovered reads located similarly as the published/reanalyzed peaks with respect to the TAL1 target genes, we analyzed the position of the peaks with respect to different gene parts (Intergenic, Upstream of transcription start sites [3 kb], 5′-UTR, Exon, Intron, 3′-UTR, 3′ of gene [3 kb]). The newly-identified peak regions were mainly located in intergenic and intronic regions (Fig. 7a), consistent with a previous observation of TAL1's preferential binding in these regions^18^. However compared to published and reanalyzed peaks, the proportion of recovered peaks was at least 13% more in intergenic regions and 10% less in intronic regions (Fig. 7a). The distribution of the proportions of peaks for the recovered, reanalyzed and published peaks was however similar in 3′ UTR, 5′ UTR, downstream, exon and promoter regions. Peak density distribution (kernel density distribution of peaks- a measure of read enrichment near a particular gene part) showed a higher local density near transcription start sites (TSS) for all the three categories, albeit with reduced density for the recovered peaks (Fig. 7b). This type of distribution showing enrichment of reads near TSS was previously observed in several TF binding patterns in humans as reported in the ENCODE project^1^ and in a previous study^18^.

To determine the functional categories of the newly-identified TAL1 target genes, we assigned Gene Ontology (GO) terms. Consistent with the reported role of TAL1 in leukaemogenesis by disrupting T-cell differentiation and promoting expression of anti-apoptotic genes^21^, the three main categories identified corresponded to T-cell activation, T-cell differentiation, and apoptosis (Supplementary Table S2). However, only genes associated with the recovered peaks showed a significant over-representation of GO terms corresponding to pentose phosphate metabolic processes and sensory perception. Novel TAL1 target genes associated with glucose metabolism and the Pentose Phosphate Pathway (PPP) were identified: Ribose-5-Phosphate Isomerase A (RPIA), Glucose-6-Phosphate Isomerase (GPI), and 6-Phosphogluconolactonase (PGLS). The PPP is an important metabolic pathway that is related with cancer, specifically in augmenting tumor proliferation by supplying tumor cells with NADPH^22^,^23^; modulation of metabolic enzymes involved in both oxidative and nonoxidative PPP^24^; and modulation of glycolytic flux through regulation of the glycolysis rate-limiting enzyme Glycolysis Phosphofructokinase-1 (PFK-1)^24^. Additionally, genes involved in fatty acid metabolism (Enoyl-CoA Delta Isomerase 2 [ECI2] and Prostaglandin E Synthase 2 [PTGES2]) were identified as TAL1 targets, further highlighting the role of metabolism in cancer, long observed by Warburg^25^ and confirmed in recent studies^22^,^24^,^26^. Furthermore, the discovery of several genes in the olfactory reception and/or sensory perception (OR11H12, OR2T1, OR4A47, OR4A5, OR4C12, OR4C45, OR4C46, OR4F16, OR9G4, OR9G9) suggests a new role of TAL1 yet to be investigated.

### Sequence complexity of newly-identified TAL1 binding regions

To investigate possible reasons for why the observed recovered peaks were missed in the published datasets, the characteristics of the enriched regions of the three categories of published, reanalyzed and recovered datasets were further studied. For this purpose, we determined the genome complexity of the newly-identified TAL1 binding regions.

**Figure 8:**
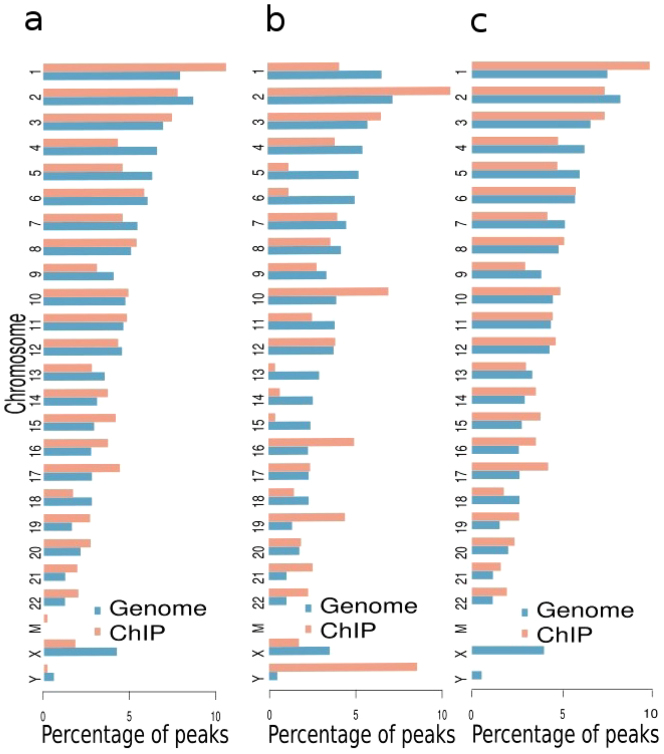
Chromosomal distribution of reanalyzed (a), recovered (b) and published (c) peaks. A significant proportion of recovered peaks are located on the Y chromosome (panel b).

Firstly, the distribution of peak regions on different chromosomes was determined for the three categories of binding profiles (published, reanalyzed and recovered binding profiles) by establishing the location of each peak in the human chromosomes. While all the three categories of reanalyzed, recovered and published peaks exhibited comparable proportions of peaks across most chromosomes (Fig. 8a,b,c), significantly higher proportion of peaks were located on the Y chromosome in the recovered peaks category (10.3%, Fig. 8b) compared to the proportion of peaks on chromosome Y in the reanalyzed peaks category (0.2%, Fig. 8a). The original TAL1 study^18^, however, did not report the proportion of peaks on the X and Y chromosomes (Fig. 8c).

Secondly, in order to study the genome complexity of the newly identified TAL1 binding sites, we identified repeat elements as markers of low sequence complexity in the new binding sites using RepeatMasker (http://www.repeatmasker.org/). Of the 597 newly-identified binding sites, 212 (35.5%) contained at least one low-complexity element, including simple repeats and satellites, compared to 5% of published and 2% of reanalyzed peaks (Supplementary Table S3, Sections 4, 5 and 6 of the Supplementary Information), suggesting the potential role of genome complexity in success of alignment of short reads.

## Discussion

The combination of computational analyses and experimental validation revealed that a high proportion of unmapped ChIP-Seq reads were mappable after being subjected to a taxonomic classification and subsequent remapping using SHRiMP, a short read aligner capable of mapping reads to highly polymorphic regions of the genome. We discovered that these previously ignored reads, also reported in ChIP-Seq experiments conducted with various modified histones^27^,^28^,^29^,^30^ harbor key information that showed novel aspects important for the reconstruction of gene regulatory grids. By focusing on the human TAL1 TF, a set of previously unaligned reads were shown to have mappable properties and have been used for identification and validation of novel TAL1 binding sites, revealing important characteristics on how this TF can regulate cell fate determination, apoptosis, and potential novel role in sensory perception. Our results suggest that re-evaluation of previously unaligned reads from past ChIP-Seq experiments can enhance construction of gene regulatory grids and networks.

## Methods

### Raw reads preprocessing and genome alignment

ChIP-Seq reads were obtained from the short read archive (SRA) repository corresponding to *A. thaliana*^8^,^9^,^31^,^32^, *D. melanogaster*^7^,^33^, *C. elegans*^2^,^34^, *H. sapiens*^18^,^35^,^36^,^37^,^38^,^39^ and *Z. mays*^40^,^41^ TF binding studies. Reads were first submitted to a preprocessing step that involved determination of sequence quality and filtering of adapter sequences using the short read quality control software fastqc (http://www.bioinformatics.babraham.ac.uk/projects/fastqc/) and FASTX- ToolKit (http://hannonlab.cshl.edu/fastx_toolkit/). Preprocessed reads were subsequently aligned to their respective reference genomes using Bowtie 0.12.7^14^ in a multi-thread mode running on a multicore cluster, allowing three mismatches (see Section 3 of the Supplementary Information for a complete set of alignment parameters and versions of the reference genomes). Reads from datasets that showed presence of potentially legitimate reads after taxonomic classification were realigned back to their reference genomes using SHRiMP version 2.2.3^19^ (see Section 3 of the Supplementary Information for alignment parameters).

### Receiver Operating Characteristic Analysis

To evaluate the accuracy of several alignment programs, one million 75 bp short reads were simulated from the maize genome using a python script wgsim (https://github.com/lh3/wgsim). Simulated reads were subsequently aligned to the maize reference genome using Novoalign version 3.01, Stampy version 1.0.23, BWA version 0.7.10, Bowtie2 version 2.1.0, Bowtie version 0.12.7 and SHRiMP version 1.3.2. Each programs' default alignment parameters were used in alignment. True positive rate (sensitivity) and false positive rate (1 - specificity) were determined from the aligned reads and represented in a Receiver Operating Characteristic (ROC) curve.

### Short Read Clustering

Reads were clustered using UCLUST^42^ based on an assigned similarity score of 75%, implying that sequences that exhibited a similarity of 75% or more were grouped in one cluster. Representative sequences from each cluster, referred to as cluster seeds, were then submitted to nucleotide database search as query sequences.

### Nucleic Acid Database Search and Taxonomic Classification

NCBI's pre-formatted nucleotide (nt) database was downloaded to a local compute cluster for ease of BLAST search. The nt database search was carried out using NCBI's BLAST stand-alone tool (http://www.ncbi.nlm.nih.gov/books/NBK1763/) in a multi-thread mode and the BLAST results generated in BLASTXML format. This was followed by assignment of the unaligned query reads into their respective Operational Taxonomic Units (OTU) based on their BLAST hits/results using an integrated Bio4j and Metagenomics7 (MG7)^43^ system, an open source system for massive analysis of sequences from metagenomics samples. MG7 carries out taxonomic classification of short reads by associating reads and their blast hits with NCBI's taxonomy tree and taxon GI index. We also carried out a nucleotide database search and taxonomic classification of a subset of Arabidopsis genome-simulated and aligned reads in order to serve as a positive control. As expected, almost all the reads in these control datasets were assigned to the same taxonomic units as the read source.

### Chromatin Immunoprecipitation qPCR (ChIP-qPCR) Experiments

Jurkat cells were cultured as described in Ref. 18. Anti-TAL1 Ab (sc-12984) and normal goat IgG (sc-2028) were purchased from Santa Cruz Biotechnology. ChIP experiments were carried out following established protocols^44^, except that DNA was purified by PCR QIAGEN purification kit. For qPCR, DNA was quantified with SYBR R Green PCR Master Mixes (Applied Biosystems). The enrichment of each peak is expressed as the fold enrichment relative to a mock ChIP performed with normal IgG.

### ChIP-Seq Peak Detection

TF enrichment was determined using MACS version 1.4.2, a peak calling software that models genome-wide short read alignment densities by a Poisson distribution^20^. Two sets of ChIP-Seq peaks were identified: (i) from alignment files (in BAM format) obtained from all preprocessed TAL1 reads (referred to as reanalyzed peaks); and (ii) from alignment files obtained from previously unaligned but recovered TAL1 reads (referred to as recovered peaks). A full set of peak-calling parameters used in analysis can be found in Section 3 of the Supplementary Information.

### Statistical Analyses

To determine if there is a significant difference in mappability of reads with different read lengths, a Chi-square test for equality of proportions was performed. In addition, to determine whether TAL1 enrichment (determined by ChIP-qPCR) is statistically significant in the recovered peaks compared to a set of negative controls, the distribution of enrichment was first assumed to be Gaussian. Then, the difference in the means of enrichment between the ChIP peak and the controls was estimated using t-test, assuming unequal variances between samples (Welch's t- test). Statistical tests were performed on the R Statistical Computing environment (http://www.r-project.org/).

## Author Contributions

W.Z.O., M.K.M.G., A.Y. and E.G. designed the research. W.Z.O. analyzed the data. P.P.T. contributed analytic tools. W.L. and A.I.D. performed experiments. W.Z.O. and E.G. wrote the paper. All authors read and approved the final manuscript.

## Supplementary Material

Supplementary Information supplementary information

Supplementary Information Supplementary File 1

Supplementary Information Supplementary File 2

## Figures and Tables

**Figure 1:**
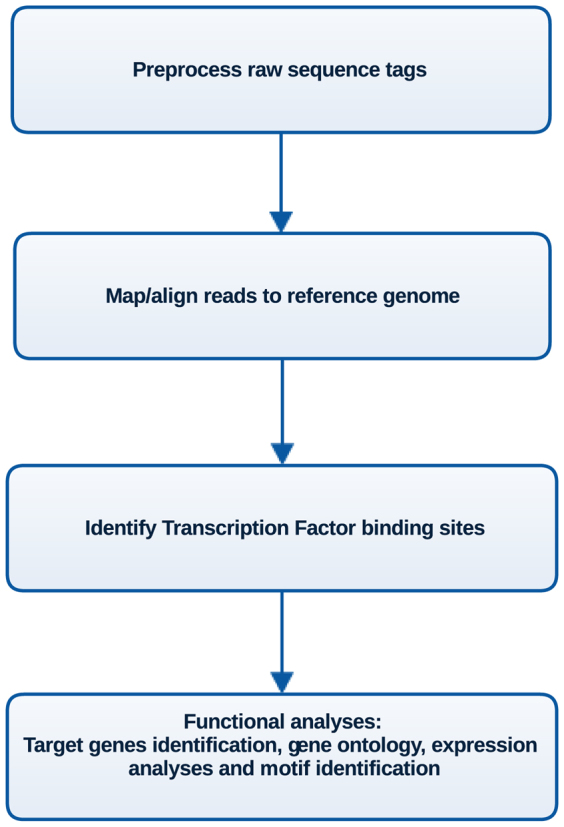
Typical ChIP-Seq analysis pipeline.
