# Supplementary material for: Important biological information uncovered in previously unaligned reads from chromatin immunoprecipitation experiments (ChIP-Seq)
Source: Sci Rep. 2015 Mar 2;5:8635. doi: 10.1038/srep08635 (PMC4345404; doi:10.1038/srep08635)
Supplement: Supplementary Information [file srep08635-s1.pdf]

Supplementary Information for

Important biological information uncovered in previously unaligned reads from chromatin immunoprecipitation experiments (ChIP-Seq)

Wilberforce Zachary Ouma[1,2] , Maria Katherine Mejia-Guerra[1,2] , Alper Yilmaz[3] , Pablo Pareja Tobes[4], Wei Li[5,6] , Andrea I. Doseff[5,6] and Erich Grotewold[2,5*]

1. Molecular, Cellular and Developmental Biology (MCDB) Graduate Program, The Ohio State University, Columbus, OH., USA.

2. Center for Applied Plant Sciences (CAPS), The Ohio State University, Columbus, OH., USA

3. Department of Bioengineering, Yildiz Technical University, Istanbul, Turkey.

4. Oh no sequences! Research group, Era7 Information Technologies SLU, Granada, Spain

5. Department of Molecular Genetics, The Ohio State University, Columbus, OH., USA

6. Department of Internal Medicine, Division of Pulmonary, Allergy, Critical Care, and Sleep, Heart and Lung Research Institute, The Ohio State University, Columbus, OH., USA

* Correspondence:

Email: grotewold.1@osu.edu

Center for Applied Plant Sciences (CAPS), The Ohio State University, Columbus, OH., USA

This supplementary information document includes:

1. A description of Supplementary Files
2. Supplementary Figure S1 to S9
3. Supplementary Table S1 to S3
4. Supplementary Sections 3, 4, 5 and 6

Description of Supplementary Files

Supplementary File 1 as XLS Sheet 1 — Short Read Archive (SRA) accession numbers and dataset description for *A. thaliana*

Supplementary File 1 as XLS Sheet 2 — Short Read Archive (SRA) accession numbers and dataset description for *H. sapiens*

Supplementary File 1 as XLS Sheet 3 — Short Read Archive (SRA) accession numbers and dataset description for *D. melanogaster*

Supplementary File 1 as XLS Sheet 4 — Short Read Archive (SRA) accession numbers and dataset description for *C. elegans*

Supplementary File 2 as XLS file — Genomic locations of TAL1 recovered peaks (Sheet 1); Genomic locations and target genes of validated TAL1 peaks (Sheet 2)

Supplementary Section 3 — Commands and description of parameters for running Bowtie, SHRiMP & MACS programs; versions of reference genomes used in analysis

Supplementary Section 4 — Published peaks repeat elements

Supplementary Section 5 — Reanalyzed peaks repeat elements

Supplementary Section 6 — TAL1 recovered peaks repeat elements

Supplementary Figures

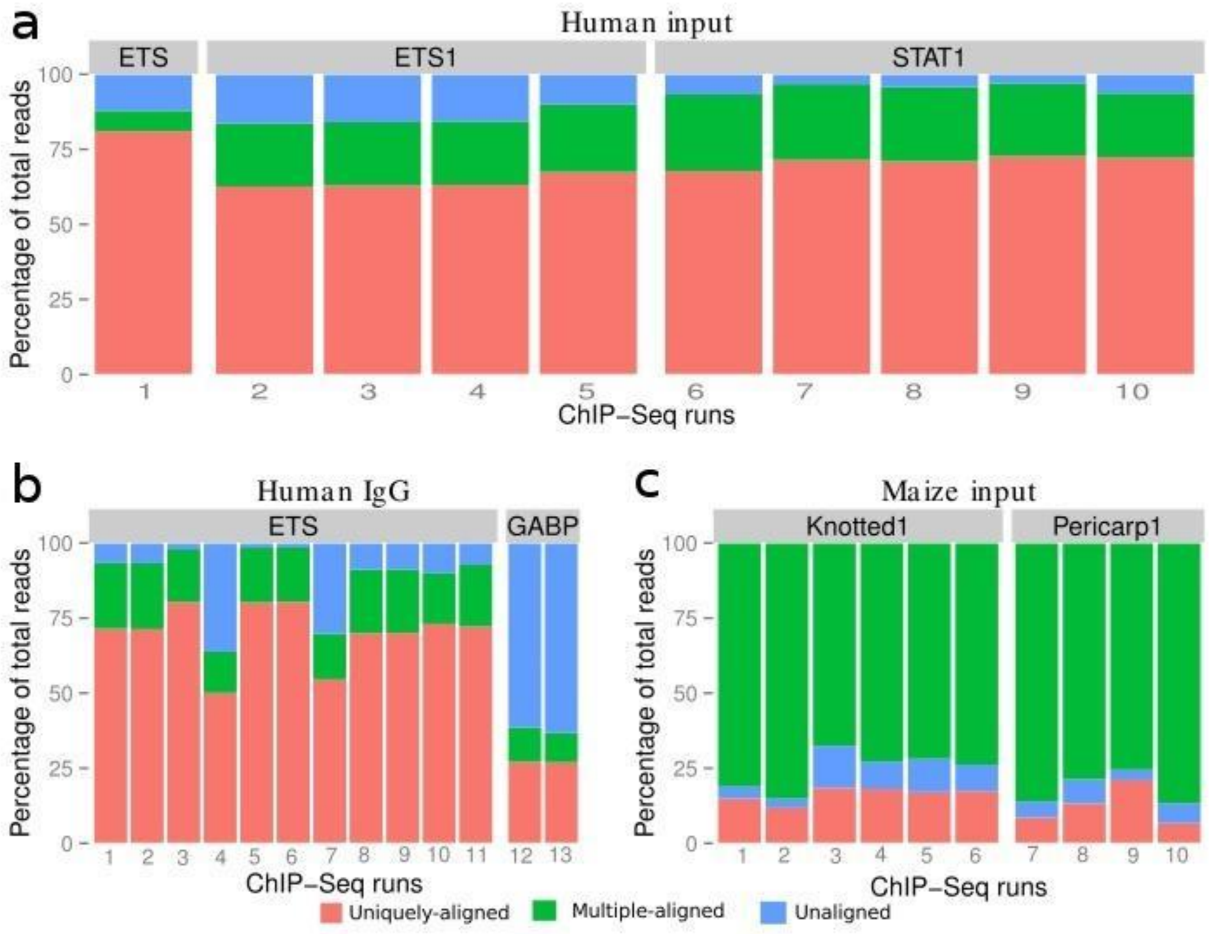

Supplementary Figure S1: Alignment proportions of control ChIP-Seq datasets. Each bar represents a ChIP-Seq run; several runs constitute an experiment for determining binding patterns of one transcription factor. Runs have been grouped into their respective TFs

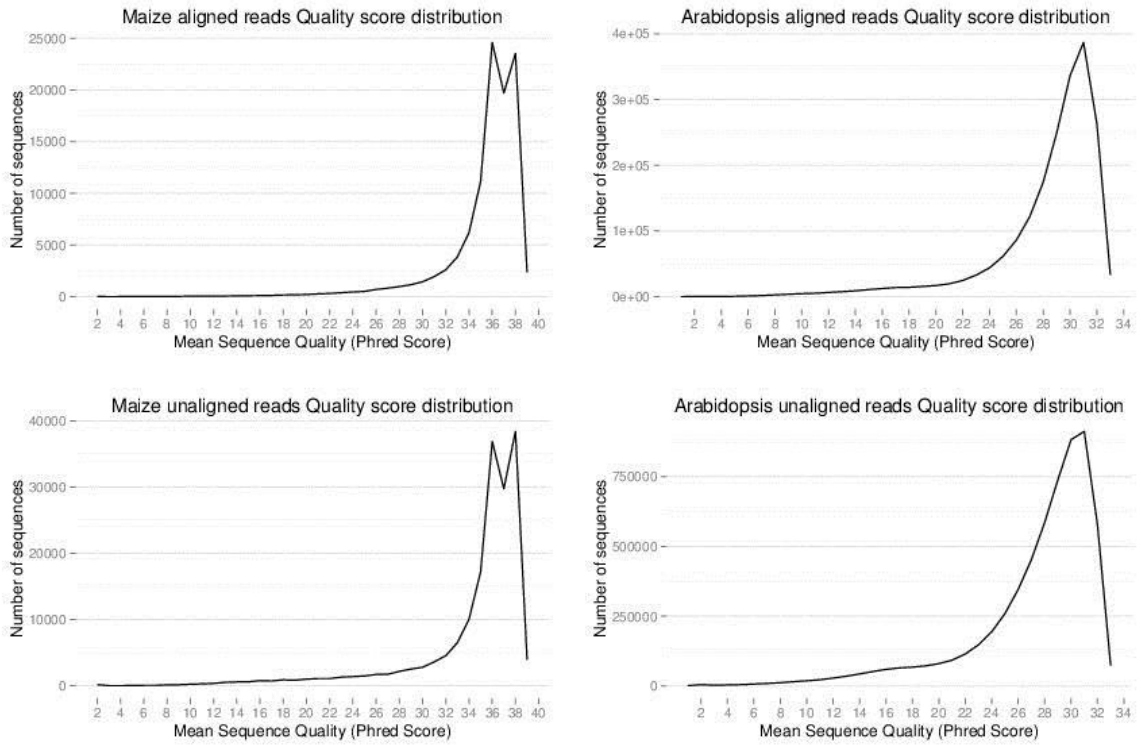

Supplementary Figure S2: Quality scores for maize and Arabidopsis ChIp-Seq reads. Both aligned and unaligned reads exhibit similar quality scores.

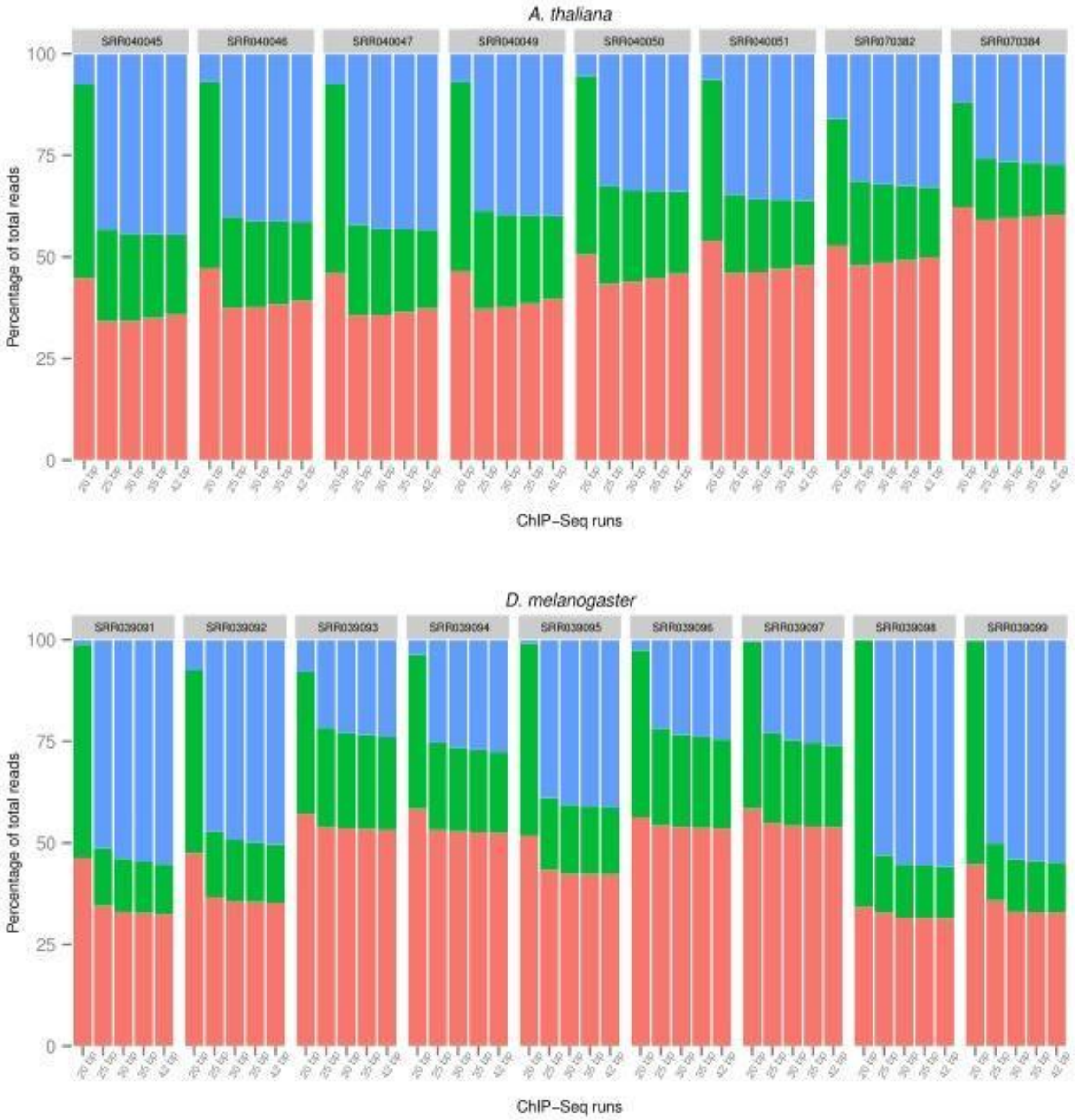

Supplementary Figure S3: Proportions of uniquely aligned, unaligned and multiple-aligned reads for *A. thaliana* and *D. melanogaster* trimmed datasets. Each facet is a ChIP-Seq run in which each bar represents a run whose reads have been trimmed to a particular read length shown on the x-axis.

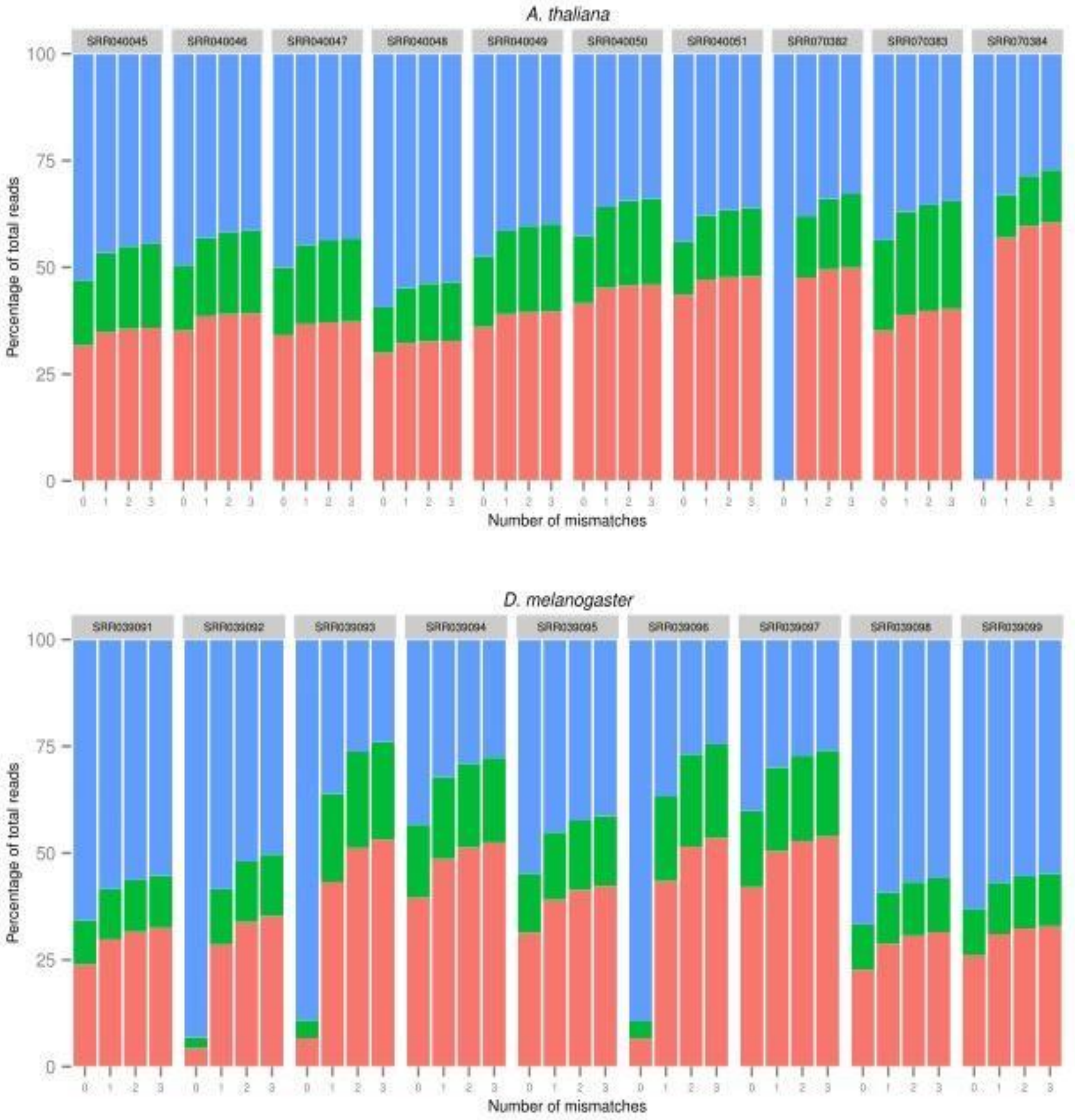

Supplementary Figure S4: Proportions of aligned, unaligned and multiple-aligned reads for different mismatches allowed in alignment process. Each facet is a ChIP-Seq run in which each bar represents a run whose reads have been aligned using the number of allowed mismatches showed on the x-axis.

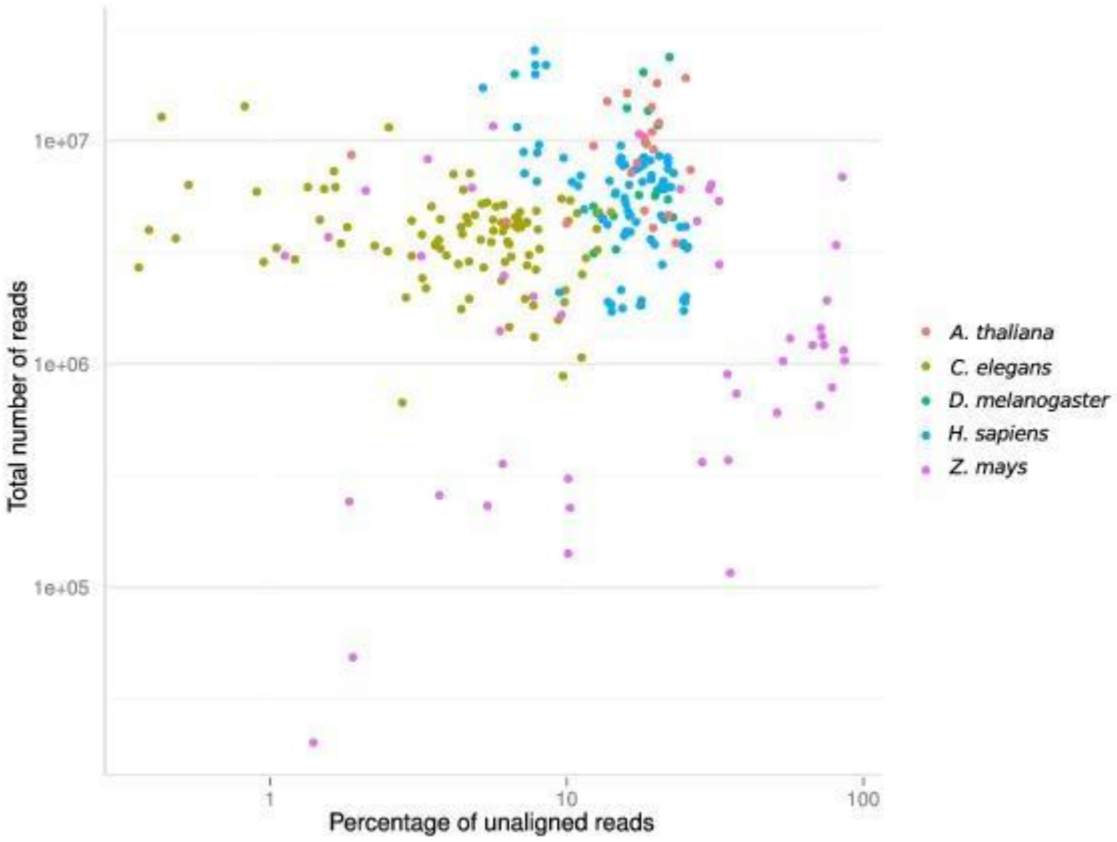

Supplementary Figure S5: Number of reads in a ChIP-Seq dataset as a function of proportion of unaligned reads from the same dataset. Lack of correlation suggests that sequencing more reads does not guarantee increased read mappability.

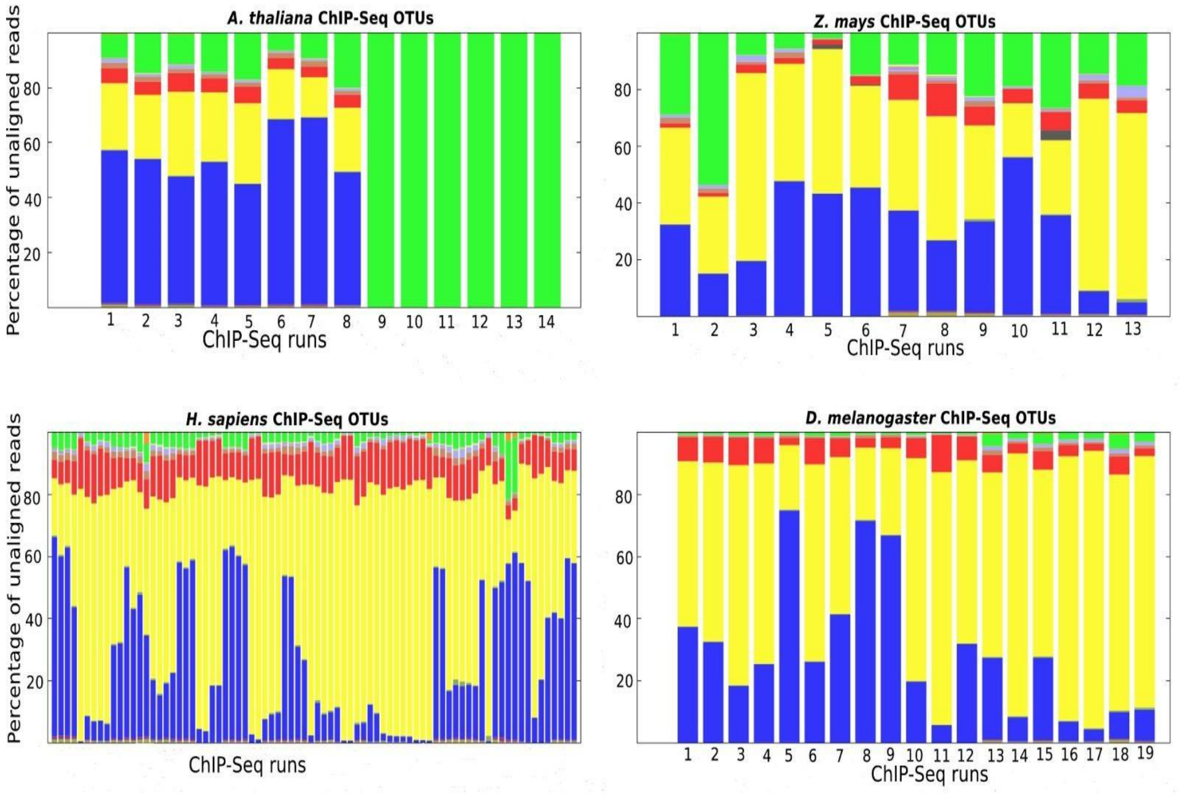

Supplementary Figure S6: Taxonomic classification of previously unaligned reads from *A. thaliana*, *Z. mays*, *H. sapiens*, and *D. melanogaster* datasets. Taxonomic classification reveals presence of bacterial (blue), metazoan (yellow) and plant derived (green) sequences. The Arabidopsis runs 9-14 correspond to Arabidopsis genome-simulated and aligned reads reads subjected taxonomic classification as a positive control (See Supplementary File 1 Sheets 5, 6 & 7 for the accession numbers corresponding to each number in the figure).

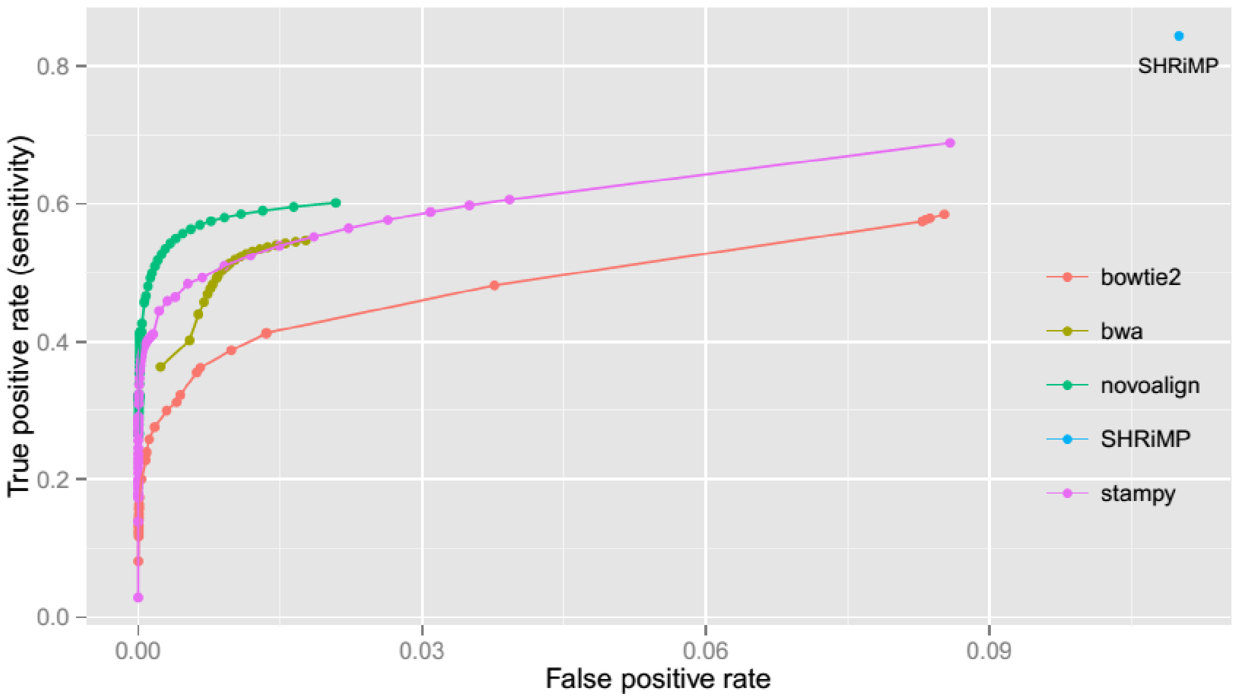

Supplementary Figure S7: A Receiver Operating Characteristic Curve illustrating the trade-off between true positive and false positive rates of selected next generation sequencing alignment programs

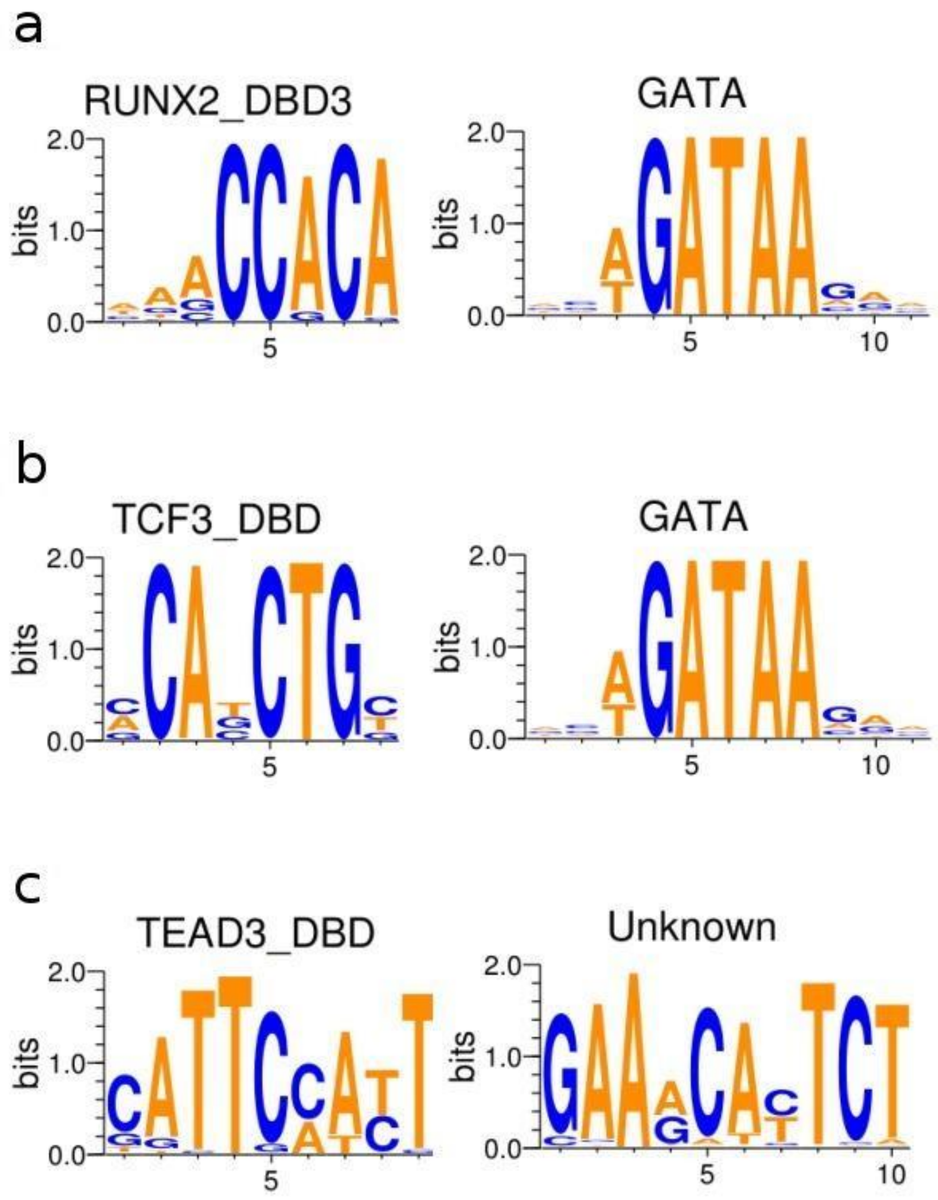

Supplementary Figure S8: Motifs enriched in published (a), reanalyzed (b) and recovered (c) peaks. Statistically over-represented motifs were identified using the *de novo* motif discovery tool MEME.

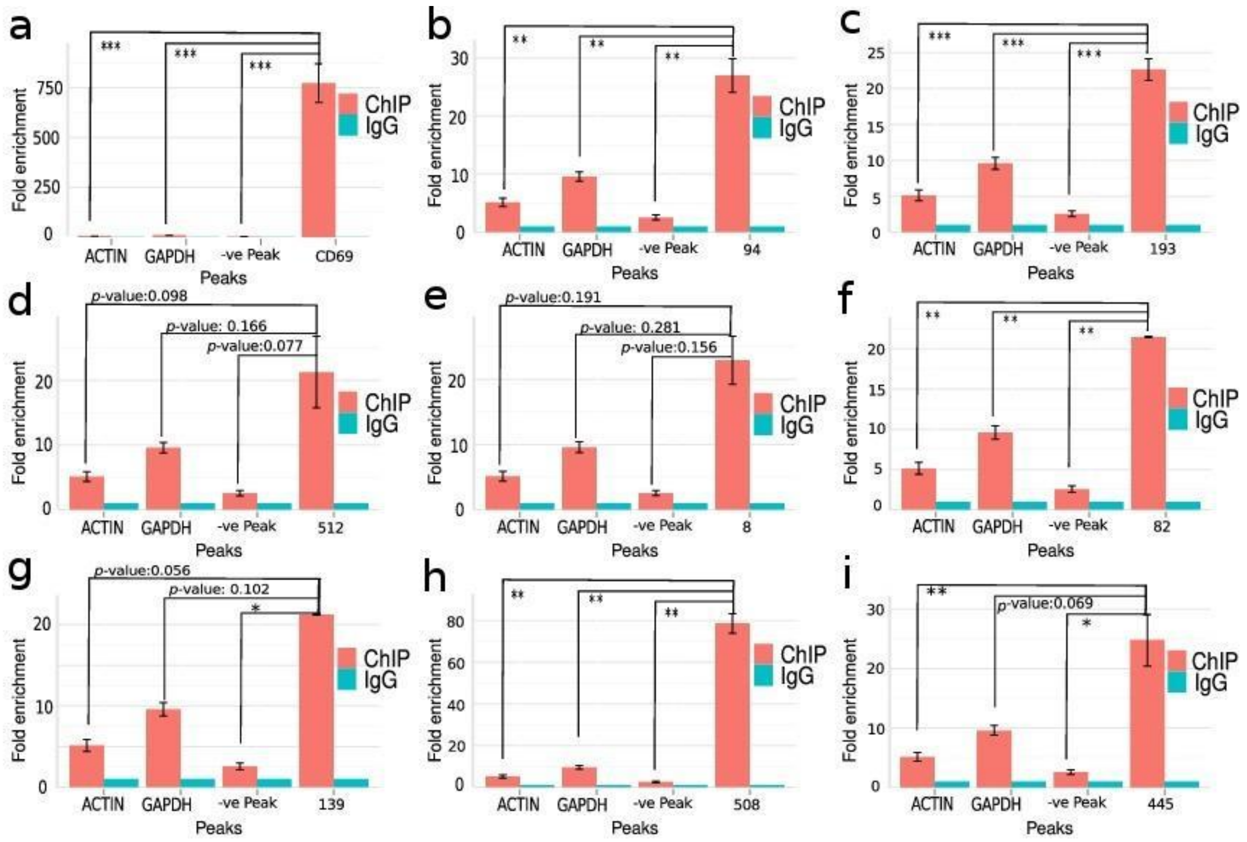

Supplementary Figure S9: ChIP-qPCR validation of selected recovered TAL1 peaks. Each bar represents fold enrichment of peaks relative to IgG. Error bars represent standard error. Statistical significance is as a result of t-test between the validated peak enrichment and the negative controls of ACTIN, GAPDH, and negative peak; n=3. Actual p-value is indicated when > 0.05, otherwise p-value < 0.05 indicated by *; < 0.01 by **; and < 0.001 by ***;- ve: negative.

Supplementary Tables

Supplementary Table S1: Average GC content for the five genomes analyzed.

**Genome**	**GC content, %**
H. sapiens	40.38
C. elegans	35.44
D. melanogaster	41.51
Z. mays	46.81
A. thaliana	36.05

Supplementary Table S2: GO term analysis of genes closest to TAL1 published, reanalyzed and recovered peaks

**Published peaks GO terms:**	**$-\log_{10}(p\text{-value})$**
T- cell differentiation	16.36
T-cell activation	16.09
T-cell receptor signaling pathway	13.41
**Reanalyzed peaks GO terms:**	
T-cell activation	18.74
T-cell differentiation	18.49
T-cell receptor signaling pathway	14.82
**Recovered peaks GO terms:**	
Pentose-phospahate shunt pathway	27.47
Sensory perception	17.12
Olfactory transduction	13.39
Apoptosis	2.38
T-cell activation	1.42

Supplementary Table S3: Repeat elements identified published (A), reanalyzed (B) and recovered peaks (C)

A: Published peaks

	Number of elements	Length occupied, bp	Percentage of sequence, %
**Satellites**	0	0	0.00
**Simple repeats**	93	3888	0.50
**Low complexity**	22	941	0.12

B: Reanalyzed peaks

	Number of elements	Length occupied, bp	Percentage of sequence, %
**Satellites**	2	1144	0.17
**Simple repeats**	65	4476	0.67
**Low complexity**	9	401	0.06

C: Recovered peaks

	Number of elements	Length occupied, bp	Percentage of sequence, %
**Satellites**	121	61773	18.56
**Simple repeats**	188	31934	9.59
**Low complexity**	55	7685	2.31

Section 3

Bowtie alignment command and parameters:

/nfs/15/osu4902/bowtie -p 10 -q -v 3 -m 1 -S --best --strata --chunkmbs 128 -t /nfs/15/osu4902/bowtie_db/a_thaliana SRR016811.fastq ../sam/SRR016811.sam --un ../unaligned/SRR016811_unaligned.fq --al ../aligned/SRR016811_aligned.fq --max ../aligned/SRR016811_multiple_align.fq

Parameters:
-p <integer>: number of processor core threads used in alignment
-q: input files are in FASTQ format
-v <integer>: report end-to-end hits with less than or equal v mismatches
-m <integer>: maximum number of multiple alignments allowed
-S: write hits in SAM format
--best --strata: only hits in best stratum are reported
--chunkmbs <integer>: maximum megabytes for RAM for best first search frames
-t: print wall-clock time taken by search phases
--un <filename>: write unaligned reads to <filename>
--al <filename>: write aligned reads to <filename>
--max <filename>: write multiple-aligned reads to to <filename>

SHRiMP alignment command and parameters:

/nfs/15/osu4902/applications/SHRiMP_2_2_0/bin/gmapper-ls --qv-offset 33 -L /nfs/proj14/PAS0107/reads_recovery/hg18-20gb-12_12_12_12seeds-"$i"of3-ls --strata --max-alignments 1 -N 12 --un SRR054882_homo_sapiens_${i}of3_unaligned.fq -E -Q SRR054882_homo_sapiens_sequence_reads.fq >SRR054882_homo_sapiens_${i}of3.sam

Parameters:
--qv-offset <integer>: interpret quality values in fastq input as PHRED+<integer>
-L: reference genome idex
--strata: print only best scoring hits
--max-alignments <integer>: maximum alignment per read
-N <integer>: number of processor core threads
--un <filename>: write unaligned reads to <filename>
-E: output SAM format
-Q: input reads are in FASTQ format

MACS peak calling command and parameters:

macs14 -t ChIP.bam -c Control.bam -f BAM -g h -n test -w -p 1e-5 -B

Parameters:
-t: ChIP-seq treatment files
-c: control files
-f: format of alignment file
-g: effective genome size
-n: experiment name
-w: Whether or not to save extended fragment pileup into a wiggle file
-p: Pvalue cutoff for peak detection
-B: Whether or not to save extended fragment pileup at every bp into a bedGraph file

Genome versions:
A. thaliana: TAIR9 genome release
Z. mays: B73 version 2 (ZmB73_RefGen_v2)
H. sapiens: version 19 (GRCh37/hg19)
C. elegans: version WS200
D. melanogaster: release 5.22

Section 4

```
==================================================
file name: GSM614003_jurkat.tal1_tab_hg19.bed.fasta
sequences:          2238
total length:     771448 bp  (771448 bp excl N/X-runs)
GC level:         48.05 %
bases masked:       4829 bp ( 0.63 %)
==================================================
               number of      length   percentage
               elements*    occupied  of sequence
--------------------------------------------------
SINEs:                0            0 bp    0.00 %
      ALUs            0            0 bp    0.00 %
      MIRs            0            0 bp    0.00 %

LINEs:                0            0 bp    0.00 %
      LINE1           0            0 bp    0.00 %
      LINE2           0            0 bp    0.00 %
      L3/CR1          0            0 bp    0.00 %

LTR elements:         0            0 bp    0.00 %
      ERVL            0            0 bp    0.00 %
      ERVL-MaLRs      0            0 bp    0.00 %
      ERV_classI      0            0 bp    0.00 %
      ERV_classII     0            0 bp    0.00 %

DNA elements:         0            0 bp    0.00 %
     hAT-Charlie      0            0 bp    0.00 %
     TcMar-Tigger     0            0 bp    0.00 %

Unclassified:         0            0 bp    0.00 %

Total interspersed repeats:        0 bp    0.00 %


Small RNA:            0            0 bp    0.00 %

Satellites:           0            0 bp    0.00 %
Simple repeats:      93         3888 bp    0.50 %
Low complexity:      22          941 bp    0.12 %
==================================================

* most repeats fragmented by insertions or deletions
  have been counted as one element


The query species was assumed to be homo
RepeatMasker version open-3.3.0 , default mode

run with rmblastn version : 2.2.27+
The query was compared to classified sequences in ".../RepeatMasker.lib"
RepBase Update 20110419-min, RM database version 20110419-min
```

Section 5

```
==================================================
file name: SRR070589-original_peaks.bed.fasta
sequences:          3344
total length:     666435 bp  (666435 bp excl N/X-runs)
GC level:         48.99 %
bases masked:       5264 bp ( 0.79 %)
==================================================
               number of      length   percentage
               elements*    occupied  of sequence
--------------------------------------------------
SINEs:                0            0 bp    0.00 %
      ALUs            0            0 bp    0.00 %
      MIRs            0            0 bp    0.00 %

LINEs:                0            0 bp    0.00 %
      LINE1           0            0 bp    0.00 %
      LINE2           0            0 bp    0.00 %
      L3/CR1          0            0 bp    0.00 %

LTR elements:         0            0 bp    0.00 %
      ERVL            0            0 bp    0.00 %
      ERVL-MaLRs      0            0 bp    0.00 %
      ERV_classI      0            0 bp    0.00 %
      ERV_classII     0            0 bp    0.00 %

DNA elements:         0            0 bp    0.00 %
     hAT-Charlie      0            0 bp    0.00 %
     TcMar-Tigger     0            0 bp    0.00 %

Unclassified:         0            0 bp    0.00 %

Total interspersed repeats:        0 bp    0.00 %


Small RNA:            0            0 bp    0.00 %

Satellites:           2         1144 bp    0.17 %
Simple repeats:      65         4476 bp    0.67 %
Low complexity:       9          401 bp    0.06 %
==================================================

* most repeats fragmented by insertions or deletions
  have been counted as one element


The query species was assumed to be homo
RepeatMasker version open-3.3.0 , default mode

run with rmblastn version : 2.2.27+
The query was compared to classified sequences in ".../RepeatMasker.lib"
RepBase Update 20110419-min, RM database version 20110419-min
```

Section 6

```
==================================================
file name: SRR070589_recovered_peaks.xls.bed.fasta
sequences:            594
total length:      332838 bp  (332697 bp excl N/X-runs)
GC level:          43.97 %
bases masked:       89271 bp ( 26.82 %)
==================================================
               number of      length   percentage
               elements*    occupied  of sequence
--------------------------------------------------
SINEs:                0            0 bp    0.00 %
      ALUs            0            0 bp    0.00 %
      MIRs            0            0 bp    0.00 %

LINEs:                0            0 bp    0.00 %
      LINE1           0            0 bp    0.00 %
      LINE2           0            0 bp    0.00 %
      L3/CR1          0            0 bp    0.00 %

LTR elements:         0            0 bp    0.00 %
      ERVL            0            0 bp    0.00 %
      ERVL-MaLRs      0            0 bp    0.00 %
      ERV_classI      0            0 bp    0.00 %
      ERV_classII     0            0 bp    0.00 %

DNA elements:         0            0 bp    0.00 %
     hAT-Charlie      0            0 bp    0.00 %
     TcMar-Tigger     0            0 bp    0.00 %

Unclassified:         0            0 bp    0.00 %

Total interspersed repeats:        0 bp    0.00 %


Small RNA:            0            0 bp    0.00 %

Satellites:         121        61773 bp   18.56 %
Simple repeats:     188        31934 bp    9.59 %
Low complexity:      55         7685 bp    2.31 %
==================================================

* most repeats fragmented by insertions or deletions
  have been counted as one element


The query species was assumed to be homo
RepeatMasker version open-3.3.0 , default mode

run with rmblastn version : 2.2.27+
The query was compared to classified sequences in ".../RepeatMasker.lib"
RepBase Update 20110419-min, RM database version 20110419-min
```
